# A method for metabolomic sampling of suspended animal cells using fast filtration

**DOI:** 10.1186/1753-6561-5-S8-P93

**Published:** 2011-11-22

**Authors:** Martin Volmer, Julia Gettmann, Sebastian Scholz, Heino Büntemeyer, Thomas Noll

**Affiliations:** 1 Institute of Cell Culture Technology, Bielefeld University, D-33615 Bielefeld, Germany

## Background

For intracellular metabolomic analyses it is of utmost importance to rapidly stop the organism's metabolism during sampling. This is to avoid false data due to residual enzymatic activity during sampling. Unlike for bacteria and yeast, there is not one generally approved protocol for metabolome sampling of suspended animal cells. A number of sampling procedures have been developed and described in literature but have not been compared in depth.

Here we describe a sophisticated sampling method for metabolome analysis of suspended animal cells using a fast filtration protocol. The main requirements for the fast filtration method were to reduce the time for quenching and cell-medium separation while reducing cell disruption to a minimum.

Additionally, the fast filtration method is compared to the other sampling methods described in literature.

## Methods

CHO DP12 cells were cultured in shaker flasks and 2 L Bioreactors to generate sample cell suspensions for the experiments.

The different quenching methods were used according to the literature. Methods tested included sampling with fast filtration [1], sampling in cold saline solution [2], sampling in cold methanol/ammonium bicarbonate (AMBIC) [3], and sampling with a microstructure heat exchanger [4]. As a reference sampling of cells using centrifugation without dedicated quenching was used.

For ^13^C-labeling experiments the cells were transferred to saline solution prior to sampling. ^13^C-labeled glucose was added to the cells followed by immediate sampling. The ratio of labeled and unlabeled glycolysis metabolites was then determined.

LDH and ATP release from the cells was measured using plate tests. The intracellular energy metabolism was investigated using HILIC columns on a LC-MS system.

## Results

The cell disruption during sampling was investigated by measurement of LDH release from the cells. It was low at 5 % and 2.5 % for fast filtration and centrifugation, respectively. No influence of the cell disruption on the measured intracellular metabolite levels was observed. The intracellular amino acid content per cell was constant for filters loaded with up to 6x10^7^ cells. Thus, indicating a good extraction efficiency with the tested cell numbers. Furthermore this suggests that no residual medium components remained on the filter.

The adenylate concentrations were measured in extracts from all tested sampling methods. These showed good correlation of ATP concentrations for all sampling methods tested. The same was true for ADP and AMP concentrations. Thus, fortifying the observation of a good extraction efficiency of cells on filters.

**Figure 1:**
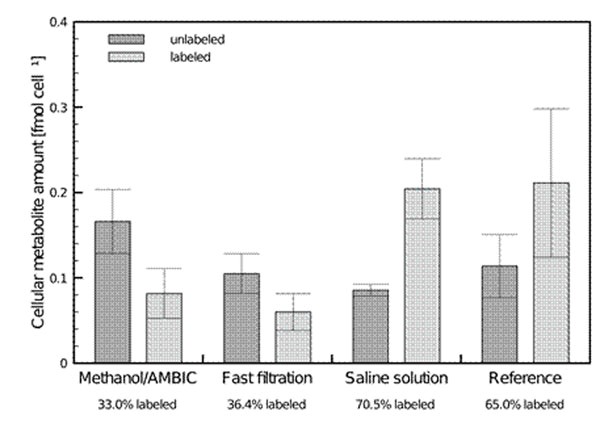
Percentage of ^13^C-labeled hexose 6-phosphate after feeding the cells with ^13^C-glucose immediately prior to sampling (N=4)

The adenylate energy charge (AEC) was used as an indicator of quenching efficiency for the comparison of the proposed method with those from literature. A significant advantage over sampling using a microstructure heat exchanger or a simple centrifugation protocol was observed. Comparison to quenching in cold methanol with ammonium bicarbonate and cold saline solution exhibited AEC values similar to quenching with fast filtration. Furthermore, experiments with isotope labeled glucose (Figure 1) exhibited only small amounts of labeled glycolysis intermediates in extracts of samples taken with fast filtration and cold methanol/AMBIC compared to saline solution quenching and centrifugation. This is most likely due to the lower temperature when sampling with methanol/AMBIC and the shorter overall sampling time when using fast filtration.

Metabolite leakage of the two methods with the best quenching efficiency was done by investigating the ATP concentration in spent washing solutions after sampling. Sampling with methanol showed ATP leakage of up to 14% whereas no leakage of ATP was observed when sampling with the fast filtration method.

## Conclusion

The results show that the fast filtration method is well applicable for metabolome sampling of suspended animal cells. The two major concerns with this method were that medium components remained with the cells after filtration and that extraction of metabolites from the filter would not be efficient [2]. Both of which could be disproved in this study.

The comparison of the different sampling methods described in literature showed that fast filtration and quenching in cold methanol/AMBIC offer the best quenching efficiency. The drawback of sampling using methanol is the metabolite leakage caused by the contact of methanol with the cell membrane. This leaves the fast filtration method as the best suited method for reliable metabolome sampling for suspended animal cells.
